# The Effect of Adenotonsillectomy on Children's Quality of life

**Published:** 2014-10

**Authors:** Ramin Zojaji, Morteza Mirzadeh, Morteza Mazloum Farsi Baf, Mostafa Khorashadizadeh, Hamid Reza Sabeti

**Affiliations:** 1 *Department of Otorhinolaryngology, Mashhad branch, Islamic Azad University, Mashhad, Iran.*; 2 *Department of Pediatrics, Mashhad branch, Islamic Azad University, Mashhad, Iran.*; 3 *General Practitioner, faculty of medicine, Mashhad branch, Islamic Azad University, Mashhad, Iran.*; 4 *Medical Student, faculty of medicine, Mashhad branch, Islamic Azad University, Mashhad, Iran.*

**Keywords:** Adenotonsillectomy, Adenotonsillar hypertrophy, Behavior, Sleep disorders.

## Abstract

**Introduction:**

Adenoid hypertrophy is the most common cause of chronic airway obstruction in children. The aim of this study was to evaluate the effect of adenotonsillectomy on sleep and behavioral disorders in children with adenotonsillar hypertrophy.

**Materials and Methods:**

In a prospective observational study, all children with an indication of adenotonsillectomy based on American Academy of Otolaryngology – Head and Neck Surgery criteria and sleep disorders referred to our otolaryngology clinic were enrolled and examined. Rutter Children’s Behavior (RCBQ) and Child Sleep Habit Questionnaires (CSHQ) were completed by the children’s parents both before and 3 months after the operation.

**Results:**

A total of 44 children (25 boys and 19 girls) with a mean (standard deviation [SD]) age of 7.86± 2.26 years and mean (SD) body mass index (BMI) of 16.37±1.35 kg/m^2^ were studied. Mean scores of RCBQ and CSHQ decreased significantly 3 months after adenotonsillectomy, and sleep habits and behavior improved significantly (P<0.05). Although there was no significant relationship between improvement of behavior and the gender, age or BMI of the children, there was a significant relationship between improvement of sleep habits and age as well as BMI (P<0.05).

**Conclusion:**

As adenotonsillectomy causes significant improvements in children`s quality of life (including sleep and behavior), it is recommended in children with adenotonsillar hypertrophy.

## Introduction

The most common cause of chronic airway obstruction in children is adenoid hypertrophy, presenting with a large spectrum of clinical signs and symptoms (1).

Lymphoid hyperplasia may occur because of repetitive infection, allergy, or unspecified reasons, and can cause partial or complete obstruction of the nasopharynx (2). Adenoids commonly occur in children from 3 to 6 years of age, and surgery is necessary when they are accompanied by obstruction and recurrent upper respiratory tract infection (3). Clinical manifestations of adenoid hypertrophy include oral respiration, snoring, and obstructive sleep apnea (OSA) in some cases. Also, the Eustachian tube may become blocked because of hypertrophic adenoids, potentially leading to recurrent otitis media or upper airway obstruction in children (4).

Apnea is defined as respiratory failure due to lack of respiratory function(central apnea) or complete airway blockage (obstructive apnea). Often, cases have symptoms of both conditions (mixed apnea) (5).

The signs of airway obstruction in children during sleep were identified almost 100 years ago, and were called OSA Syndrome (OSAS) (1). This syndrome affects 1-2% of children and can cause complications including cardiovascular, neurobehavioral, and growth disorders (6-10). OSAS in children is usually due to adenotonsillar hypertrophy. As the child matures, these complications become aggravated and lead to behavioral, learning, and cardiovascular problems, all with an incidence rate of 3% (1).

OSAS symptoms include morning lethargy, daily fatigue, and drowsiness during the day along with memory disorders, excessive weight, erratic loud snoring, periods of apnea during the night, and behavioral disorders.

The aim of this study was to investigate the effect of adenotonsillectomy on sleep and behavioral disorders in children with adenotonsillar hypertrophy.

## Materials and Methods

This prospective observational study was conducted in children with adenoid and tonsillar hypertrophy referred to the ear, nose, throat (ENT) clinic of 22-Bahman Hospital, Mashhad, Iran during the year 2011.

School-aged children with adenoids as well as tonsillar hypertrophy with an indication of adenotonsillectomy based on American Academy of Otolaryngology – Head and Neck Surgery (AAO-HNS) criteria (11) and sleep disorders were included in the study by convenience sampling. Inclusion criteria included age 6-10 years, normal intelligence quotient (IQ) (but not mentally retarded), adenotonsillar hypertrophy, having indication for adenotonsillectomy according AAO-HNS criteria, and sleep and behavior disorders. Those with obesity (body mass index [BMI]≥30), oral tumors, septal deviation, nasal polyps, and a history of previous adenotonsillectomy were excluded from the study.

The study protocol was approved by the ethics committee at Islamic Azad University and informed consent was obtained from the parents before enrollment.

The Rutter Children’s Behavior Questionnaire (RCBQ) (scale A), which is completed by the parents (usually the mother), was the instrument used for assessment of behavioral disorders among the children while the Child Sleep Habit Questionnaire (CSHQ) (12), which is again completed by parents, was applied to evaluate the children’s sleep habits and disturbances (13). 

The parental RCBQ is an 18-item questionnaire that includes descriptions of the child’s behaviors. Parents indicate whether each description ‘does not apply’, ‘applies somewhat’, or ‘definitely applies’ (12). 

RCBQ scoring is performed by summing responses given to each item from 0 to 2, where a score of 0 indicates ‘Does not apply’; 1, ‘Applies somewhat’ and 2, ‘Certainly applies’. A high score indicates behavior-adjustment problems. 

A RCBQ result was considered “normal” if the score was below the 80^th^ percentile; “moderate problem” if the score was between the 80^th^ and 95^th^ percentiles; and “severe problem” if the score was above the 95th percentile (12). 

The inter-rater and retest reliability for this test has been reported to be relatively good, although lower than that of the teachers’ questionnaire (inter-rater reliability [r= 0.64] and retest reliability [r= 0.74]) (14). 

RCBQ has been used in various studies in Iran (15-17) and its validity and reliability has been demonstrated previously (Cronba- ch's alpha: 85%) (15). 

CSHQ is a 33-item retrospective parent questionnaire that has been developed for assessment of sleep habits in school-aged children. Questionnaire items are categorized in eight sections of sleep domains including bedtime resistance, sleep onset delay, sleep duration, sleep anxiety, night waking, sleep-disordered breathing, parasomnias, and daytime sleepiness (13). Parents recall their child’s sleep behaviors during the previous week. The questionnaire total scale is calculated by summing all item scores and items are rated on a three-point scale. A score of zero indicates “none” if no occurrence/week; one is indicative of “rarely” (zero to one time/week); 2, “sometimes” (two to four times/week); and 3, “usually” if the sleep behavior occurred five to seven times/week. A higher CSHQ score indicates more disturbed sleep (13). 

In this study, a total CSHQ score higher than 41 was considered as sleep disturbance (13). 

CSHQ has been used previously in Iran and its validity and reliability have been demonstrated (Cronbach's alpha:97%) (18). 

RCBQ and CSHQ were given to the parents of patients with adenotonsillar hypertrophy and completed both before and 3 months after the operation.

***Data Analysis***

Data analysis was performed using SPSS software version 18.00 for Windows. Results for categorical variables are presented as number and percentage and continuous numerical variables as mean±standard deviation (SD). Normality of data distribution was assessed using the Kolmogorov–Smirnov test. The Chi-Square test was used for comparison of categorical data. A paired t-test and linear models with repetitive measures were used for analysis of quantitative data with normal distribution, and Mann-Whitney U and Wilcoxon signed-rank tests were applied for those without normal distribution. A paired t-test was applied for comparison of daytime sleepiness and total RCBQ score before and after the study. For comparison of morning waking, night waking, sleep onset delay, and total CSHQ score before and after the study, the Wilcoxon signed-rank test was used. To compare behavioral and sleep improvement between boys and girls, the Mann-Whitney U test was used. To test correlation between improvement and age or BMI, a Pearson correlation test was applied. The significance level was set at P≤0.05.

## Results

Forty-four patients, including 25 males (56.8%) and 19 females (43.2%), with a mean (SD) age of 7.86±2.26 years (range, 4-12 years) and a mean (SD) BMI of 16.37±1.35 kg/m^2^ (range, 14-20) were included in the study between 2010 and 2011. The frequency of the main symptoms is shown in (Table.1). 

After 3 months of adenotonsillectomy, several changes were observed in sleep and behavioral scores (Table. 2). 

**Table1:** Main symptoms in children with adenotonsillar hypertrophy

**Main symptom**	**N (%)**
Snorting	15 (34.1)
Oral respiration	11 (23)
Repetitive rhinorrhea	5 (11.4)
Drowsiness	4 (9.1)
Sleep disturbances	6 (13.6)
Repetitive midnight waking up	3 (6.8)
Total	44 (100)

Mean RCBQ scores, as well as all its related items scores, decreased significantly and sleep quality improved significantly 3 months after surgery (P<0.05) (Table. 2). The average duration of broken sleep decreased significantly after surgery (paired t-test, P<0.05) (Table. 2). The mean pre-operation morning waking score, reduced and improved significantly after surgery (Wilcoxon test) (P=0.026). Mean daytime sleepiness score decreased and child drowsiness during the day reduced significantly after surgery (P=0.001) (Table. 2).

**Table2:** Comparison of pre- and post-operation sleep and behavior score changes

**Factor**	**Before surgery** **Mean±SD**	**After surgery** **Mean±SD**	**P-value**
Morning waking	7.61±2.81	6.47±2.77	0.014*
Daytime sleepiness	10.38 **±**2.90	8.68**±** 3.30	0.004*
Night waking	4.29**±**1.15	3.40**±**1.04	<0.001*
Sleep onset delay	11.75±3.207	10.61±2.126	0.004*
Total CSHQ score	25.65±6.02	22.77±6.66	<0.001*
Total RCBQ score	26.22±5.86	23.27±7.00	0.001*

* P≤0.05 significant

Behavioral status score (RCBQ score) decreased significantly and child behavior improved significantly after 3 months of adenotonsillectomy (Tables. 2,3; (P<0.001 in both cases). 

Sleep and behavioral improvement rate were not significantly different between boys and girls (Table. 3). There was no significant correlation between children’s behavioral improvement and their BMI or age (Table. 4), but there was a significant direct correlation between quality of sleep improvement and age as well as BMI (P<0.05) (Table. 4).

**Table 3:** Children behavioral and sleep improvement according to the gender (Mann-Whitney U)

	** Boys** ** N Mean± SD**		** Girls** **N Mean± SD**		**P-value**
Behavioral Improvement	25	-4.00**±**6.32	19	-1.57**±**3.06	0.322
Sleep Improvement	25	-7.24**±**13.53	19	-8.42**±**7.08	0.106

**Table 4:** Correlation of children behavioral and sleep improvement with age and BMI

**Variable**	**N**	**Pearson Coefficient **	**P-value **
Behavioral improvement & BMI	44	0.041	0.776
Behavioral Improvement & age	44	-0.111	0.473
Sleep improvement & BMI	44	0.381	0.011*
Sleep improvement & age	44	0.288	0.048*

* P≤0.05 significant

## Discussion

This study found a significant improvement in sleep quality and behavior 3 months after adenotonsillectomy in children with adenotonsillar hypertrophy. 

In contrast to our study and those of several other researchers, a study by Li et al conducted in 40 children with sleep-disordered breathing (SDB) and adenotonsillar hypertrophy showed no significant improvement in sleep apnea after adenotonsillectomy(19), with no remarkable improvement in the quality of life. This study used two polysomnographies, tests of variables of attention (TOVAs), and Child Behavior Checklists to compare findings before and 6 months after surgery (19). Many investigations reached the same results as our study in terms of promotion of sleep disorders, and some, such as Broekman et al (20), were indicative of more than 75% and 68% rehabilitation of sleep and behavioral disorders, respectively, based on questionnaire results after the operation. Also, many studies have shown that adenotonsillectomy, as the primary treatment, is successful in the treatment of sleep respiratory disorders (21), promoting sleep status and completely improving the quality of life in children with adenoid and adenotonsillar hypertrophy (P<0.0001) (22-25).

A study by Wei et al (23) included 117 consecutive children with SDB who underwent adenotonsillectomy. The Pediatric Sleep Questionnaire (PSQ) and Conners' Parent Rating Scale-Revised Short Form (CPRS-RS) were completed before and 6 months after surgery. Both sleep and behavior improved 6 months after adenotonsillectomy. The authors concluded that PSQ and CPRS-RS are useful measures for screening and following children after adenotonsillectomy (23). 

In a second Wei et al study that evaluated the long-term effects of adenotonsillectomy, parents of 44 children in the previous study again completed the same question- naires (PSQ and CPRS-RS) two years after surgery (23-26). The results showed no further sleep or behavior improvement above that of the previous assessment 6 months after surgery, although the differences were significant compared to the baseline (26). 

Mitchell  et al used the OSA-18 quality of life questionnaire and concluded that adenotonsillectomy markedly improves quality of life in children with either obstructive sleep apnea or mild SDB (27). In a second study, Mitchell et al used the Behavioral Assessment System for Children (BASC)score for evaluation and comparison of sleep and behavior changes after surgery (28).They showed a significant improve- ment in BASC score after surgery (28).

 In order to validate a clinical judgment score for children with SDB, Goldstein et al used the OSA-18, Pediatric Quality of Life Inventory (PedsQL) and Child Behavior Checklist questionnaires in 100 children 8 months after surgery and concluded that the Clinical Assessment Score-15 is useful in an office setting compared with polysomno-graphy (29).

 None of the above questionnaires were used in our study; other inventories were selected (26-29). 

Wood et al, observed that life quality improved after complete and even subtotal adenotonsillectomy (30). They found no significant difference in the rate of improvement in quality of life between patients undergoing adenotonsillectomy and subtotal reduction adenotonsillectomy. The authors concluded that subtotal reduction adenotonsillectomy can be considered a lower risk alternative to full adenotonsillectomy (30).

A study by Tauman et al showed that adenotonsillectomy significantly improves sleep and respiratory abnormalities in children with OSAS (30). They found that complete improvement of respiratory abnormalities occurs only in 25% of patients, and obesity is one of the determinants of surgery outcome (31).

In a recent randomized trial, 464 children with OSAS were randomly assigned to early adenotonsillectomy or a strategy of watchful waiting, and their outcome was assessed and compared 7 months after surgery (32). Results showed the superiority of early adenotonsillectomy over a watchful-waiting strategy through significantly more improvements in behavioral, quality of life, as well as polysomnographic findings and significantly greater symptom reduction. This study, like ours, confirms the beneficial effects of early adenotonsillectomy in children (32). Like other studies of this kind, the results of this study could be subject to potential bias. Our study results may be biased by the parents’ recollection of sleep habits because, after putting their child through an operation, parents will often be biased to score higher to justify the need for the operation. However, we tried to decrease this factor as much as possible by giving several instructions and notification to the families. 

The main limitations and weaknesses of this study include the lack of a control group, exclusion of obese children, absence of the application of the full RCBQ scale A, and use of a subjective questionnaire for assessment of the primary outcome measure. These factors should be considered in future studies. Another weakness of this study is that although the results are statistically significant, a clinical difference was not examined. This could be determined by looking at the effect size or percentage improvement. The main strength of our study is in the use of other surveys that have not been investigated in previous studies (26-29). 

However, the results of most studies using different surveys and follow-up durations have shown significant sleep and behavior improvements after adenoton- sillectomy; a finding which is consistent with ours. 

Further cohorts, including control groups and larger sample sizes, are warranted to replicate and confirm our results.

## Conclusion

In conclusion, the results of this study confirm that adenotonsillectomy could improve quality of life, including sleep quality and behavior, significantly in children with adenotonsillar hypertrophy. However, the rate of sleep improvement after surgery is significantly related to age and BMI but not gender. The rate of improvement of behavior has no significant relationship with age, gender, or BMI. Therefore, as adenotonsillectomy causes a significant improvement in children’s quality of life, it is recommended as an effective treatment when indicated in children with adenotonsillar hypertrophy.
